# Inhibition of lipid oxidation in refrigerated beef using multilayer encapsulated chitosan/gum arabic/polydextrose single-bulb garlic antioxidant

**DOI:** 10.5713/ab.250743

**Published:** 2026-04-02

**Authors:** Vivi Nuraini, Anang Mohamad Legowo, Sri Mulyani, Retno Adiwinarti

**Affiliations:** 1 Faculty of Animal and Agricultural Sciences, Universitas Diponegoro, Semarang, Indonesia; 2 Faculty of Technology and Food Industry, Universitas Slamet Riyadi, Surakarta, Indonesia

**Keywords:** Free Fatty Acid, Lipid Oxidation, Multilayer Encapsulation, Natural Antioxidant, Single-bulb Garlic, Thiobarbituric Acid Reactive Substances

## Abstract

**Objective:**

This study aims to evaluate the effectiveness of multilayer encapsulation of single-bulb garlic extract using a chitosan/gum arabic/polydextrose (MESG) system as a natural antioxidant in inhibiting oxidative damage in beef steak stored at refrigerator temperatures.

**Methods:**

The study was designed using a completely randomized factorial design. The first factor was the concentration of MESG (MESG-0%, MESG-4%, and MESG-8%), and the second factor was the observation day (day 0, 3, 6, 9, 12, and 15). The marinated meat samples were stored at chiller temperature (6°C) for 15 days, and then the samples were analyzed for antioxidant activity, thiobarbituric acid reactive substances (TBARS) values, free fatty acid (FFA), pH, and fatty acid profile.

**Results:**

Physical analysis results indicated that the MESG displayed a complex, porous structure of MESG. The addition of MESG had a positive effect on the TBARS value, FFAs, and pH. Compared to the control (MESG-0%), MESG-4% and MESG-8% presented significantly (p<0.05) lower FFA levels and TBARS, while maintaining the stability of C20:4 ω6 (arachidonic acid) and C20:2 (eicosadienoic acid) during a 15-day storage period. The results showed a potential protective effect of the encapsulation method against lipid oxidation during refrigerated storage.

**Conclusion:**

This encapsulation technology is a strategic approach for preserving natural antioxidant quality, thereby extending shelf life and maintaining the quality of meat products, while also providing a viable alternative to synthetic preservatives.

## INTRODUCTION

The preservation of highly perishable foods, especially meat and its derivative products, is a persistent challenge. While low-temperature storage can slow down the deterioration of meat, the spoilage process remains unaffected. Storage duration can affect the quality of sensory acceptance by changing the color, odor, flavor, and nutritional quality of beef and its processed products [1]. Lipoperoxidation and oxidation reduce the quality and nutritional value of meat products [2]. Oxidative reactions are the main non-microbial causes of meat deterioration during storage [3], reducing meat quality, as indicated by changes in texture, development of rancid flavors, and discoloration. The main cause of deterioration in meat is oxidative reactions.

Antioxidants, either synthetic or natural, are used to prevent oxidation and inhibit the development of undesirable sensory characteristics which decrease the nutritional quality, acceptability, and shelf-life of processed meat products, hence improving their stability [4]. In addition, they help preserve the characteristics of fresh foods by inhibiting oxidative reactions. Commonly used synthetic antioxidants include sodium erythorbate, sodium ascorbate, propyl gallate, butylated hydroxyanisole (BHA), tert-butylhydroquinone (TBHQ) and butylated hydroxytoluene (BHT), as well as curing salts such as nitrite and nitrate. All of these are associated with toxicological and carcinogenic effects [2].

Synthetic antioxidants can be replaced by natural alternatives [5], which have varying action mechanisms depending on the compound. In general, antioxidant effects are associated with their capability of eliminating reactive species, curbing autoxidation by donating hydrogen atoms to stabilize primary oxidation products, chelating metal ions (pro-oxidants), and inhibiting hydroperoxide degradation. Phenolic compounds are the main natural antioxidants; among the most important are tocopherols, flavonoids, and phenolic acids. These compounds have a strong ability to donate hydrogen and free radical scavenging capability [6].

Natural antioxidants can be derived from a range of natural sources, which must be safe, accessible, and free from flavors or aromas which can compromise meat quality. In this research, we focus on garlic due to its bioactive components, including allicin, diallyl sulfide (DAS), diallyl trisulfide (DATS), diallyl disulfide (DADS), ajoene, and 2-vinyldithiins. Reactions between allinase and allin from garlic occur when garlic undergoes several physical disruptions, e.g., injury, crushing, and cutting, resulting in the formation of allicin (diallyl thiosulfinate or 2-propenyl-2-propenethiol sulphinate) in less than ten seconds. We then narrow down our focus on single-bulb garlic extract (Temanggung variety), which exhibits an antioxidant capacity IC_50_ of DPPH 10.35 ppm and a total phenolic compound (TPC) of 61.02 mg GAE/g [7]. Despite these advantages, fresh garlic is considered unstable, and its active components are easily degraded, reducing their ability to inhibit oxidative damage in meat. Furthermore, most natural antioxidants exhibit poor aqueous solubility and are highly sensitive to processing and storage conditions. They also have low bioaccessibility and bioavailability [8]. Encapsulation is a commonly employed protection method to mitigate these weaknesses. This process, which involves coating solid, liquid, or gas materials, can protect sensitive substances from damage due to environmental factors [9].

Its effectiveness is determined by coating types, encapsulation technique, and concentration of coating materials to ensure maximum stabilization of the core material. Multilayer encapsulation balances performance, protection, and flexibility while ensuring better protection for encapsulated bioactive components. Besides, it safeguards these compounds and enables controlled release at target sites, thereby elevating their bioavailability and/or bioactivity and prolonging their antioxidant functions [10].

This study used 3 types of coating materials, namely chitosan (CH), gum arabic (GA) and polydextrose (POL). The primary layer is composed of CH, a material that possesses several advantageous properties, including biodegradability, biocompatibility, non-toxicity, and a positive charge [11]. However, its inadequate protective properties necessitate its combination with other substances. GA and POL have been shown to possess comparable properties, including elevated levels of solubility and stability. POL has been shown to exhibit high levels of bioactive compound retention and antioxidant activity, a property that is further enhanced by the emulsifying characteristics of GA, thereby facilitating the formation of the desired multilayer encapsulation [12,13]. It was necessary to modify the composition of the encapsulant in this study, due to the difference in encapsulation target conditions when compared to those of previous studies. In addition, the ingredients were substituted with food-grade components. In our case, multilayer encapsulation is expected to enhance the ability of single-bulb garlic extract as an agent to inhibit oxidative damage in meat. Once this method is combined with marination, it can synergistically support the curing process, allowing the meat to have a longer shelf life and avoid oxidative damage that can reduce its quality if being stored at refrigerator temperatures. Previous research has shown that encapsulation can protect bioactive components and therefore it can prevent damage of meat and meat-based products [14,15]. This approach advances both natural antioxidant delivery and encapsulation design, offering a scalable solution to meat spoilage with minimal synthetic additives. It could set a precedent for applying similar strategies to other perishable foods.

## MATERIALS AND METHODS

Single-bulb garlic (Temanggung variety), 99% ethanol (Merck), GA (Indo Food Chem), CH, POL (Fibersse), thiobarbituric acid (Merck), phenolphthalein indicator (Sigma-Aldrich), NaOH (Merck), citric acid-sodium citrate buffer solution (100 mM), potassium carbonate (pH of 12) and citric acid (pH of 1), FeSO_4_.7H_2_O stock solution of 10.000 μ mol/L, and ferric reducing antioxidant power (FRAP) reagent.

### Preparation of single-bulb garlic extract

Single-bulb garlic was acquired from a local farmer in Temanggung, Central Java, Indonesia, and selected by the size, quality, and health aspects of the garlic bulb. Garlic authenticity was determined through a determination test. An extraction method proposed by Nuraini et al [7]was adopted, namely crushing the garlic cloves and mixing them with ethanol: water = 1:4 (v/v), the ratio of solvent to garlic is 1:2 (w/v). The mixture was placed in an ultrasonic bath (Branson-1510) at 40 kHz for 15 min at room temperature. The process of obtaining the supernatant was through a decantation mechanism, namely sedimentation of the extract at a temperature of −9°C for 48 hrs.

### Preparation of dispersions

Only food-grade materials were employed. The encapsulation process begins with the preparation of dispersion solutions. Three dispersion solutions were prepared: CH, POL, and GA. Approximately 3 g of CH was mixed with 50 mL of garlic extract, while 7.64 g of GA and 12 g of POL were each weighed and mixed with 50 mL of citric acid-sodium citrate buffer solution. CH dispersion was made at a pH of 5.5 while POL and GA dispersions were made using a citric acid-sodium citrate buffer of 100 mM pH 6 and pH of 3.6, respectively. The resulting dispersions were stirred with a magnetic stirrer until the powders were completely dissolved. The pH was adjusted to the desired value by adding potassium carbonate (pH of 12) and citric acid (pH of 1).

### Encapsulation of single-bulb garlic extract

Garlic extract was encapsulated into a multilayer wall material consisted of GA, CH, and POL [16]. The GA and CH dispersions were mixed and stirred with a magnetic stirrer for one hr (400–800 rpm). The resulting GA/CH mixture was then blended with POL dispersion and GA/CH and POL were stirred for 30 min (400–800 rpm). The resulting GA/CH/POL sample was frozen for 24 hrs in freezer and dried in a freeze dryer (BenchTop Pro with Omnitronics) at −57°C with a vacuum pressure for 48 hrs. The sample was crushed using a mortar and pestle to make a powder. This powder was then packed into polyethylene bags, sealed, and stored in a desiccator.

### Properties of multilayer encapsulation of single-bulb garlic extract

*Antioxidant activity (ferric reducing antioxidant power method)*: A stock solution of FeSO_4_.7H_2_O at a concentration of 10,000 μmol/L was made by dissolving 2.78 grams of FeSO_4_.7H_2_O in 1,000 mL of distilled water. This solution was then diluted to concentrations of 1, 2, 3, 4, and 5 μmol/L. 1 mL the standard solutions was combined with 3 mL of FRAP reagent. The mixture was measured using a UV-Vis spectrophotometer (G10S; Thermo Fisher Scientific) at wavelengths between 588 and 598 nm. A volume of 0.1 mL of the respective extract solution was added to 3 mL of FRAP reagent, after which the mixture was transferred to a test tube for the purpose of measuring the absorbance. Concurrently, the absorption of the solution was measured using a spectrophotometer at a wavelength of 596 nm [17].

*Scanning electron microscope:* Testing was carried out using a scanning electron microscope (SEM-EDX JEOL JSM-6510LA; JEOL), which produces images of samples by scanning their surfaces with a focused electron beam. Magnifications of 1,000× and 3,000× were used.

*Laser particle size analysis:* Laser particle size analysis (LPSA) were measured using the method of Houghton et al [18] with minor modifications. The particle size analyzer (LLPA-C10; Labtron) operates using the dynamic light scattering (DLS) method with a maximum wavelength of 633 nm.

### Design of the experiment

The study was designed using a completely randomized factorial design. The first factor was the concentration of encapsulated garlic powder (0%, 4%, and 8%), and the second factor was the observation day (day 0, 3, 6, 9, 12, and 15). Multilayer encapsulated single-bulb garlic extract CH/GH/POL (MESG) was applied to beef loin cut with steak-sized portions (100 g). Each meat sample was marinated with encapsulated GA/CH/POL single-bulb garlic at concentrations of 0% (w/w) (MESG-0%), 4% (w/w) (MESG-4%), and 8% (w/w) (MESG-8%). A dry marinade method was employed. MESG was applied to the beef surface by physically dispersing the encapsulated powder at a specified dosage. The powder was gently spread over the whole surface to ensure consistent coating prior to storage and subsequent analysis. Nevertheless, the uniformity of powder distribution was not quantitatively assessed. Furthermore, the marinated beef samples were stored at chiller temperature (6°C) for 15 days. Each treatment was subjected to three replications.

### Thiobarbituric acid reactive substances

The thiobarbituric acid reactive substances (TBARS) were measured using the method of Katsanidis and Zampouni [19] with minor modifications in the sample to reagent ratio. Three g of sample (beef loin) were dissolved using 50 ml of distilled water. The sample was then placed in a 1,000 mL distillation flask and washed with 48.5 ml of distilled water, to which 1.5 mL of 4 N HCl was added. The distillation process was then carried out for 10 minutes to produce 50 mL of distillate. Then, 5 mL of the distillate was collected and 5 mL of TBA reagent (a solution of 0.02 M thiobarbituric acid in 90% glacial acetic acid) was added. All samples were heated in a 98°C water bath for 35 minutes, after which they were cooled with cold tap water. For the next stage, the test tube was cooled under running water, after which absorbance measurements were taken at a wavelength of 528 nm using a Shimadzu UV-1700 spectrophotometer (Shimadzu Europe) with distilled water set as the zero point.

### Free fatty acid

The 5 g sample (beef steak) was placed into an Erlenmeyer flask. It was then supplemented with 50 mL of ethanol 95%. The sample was heated for 10 minutes while being stirred. Once cooled, 3–5 drops of 1% phenolphthalein indicator solution were added and the sample was titrated with a standardized 0.1 N sodium hydroxide solution until the color turned pink [20]. Free fatty acid (FFA) content (% FFA) was calculated using the following formula:


(1) $$\%\;\text{FFA}=\frac{mLNaoH\times MNaOH\times MM\;\text{fatty acid}}{\text{Sample weight }(g)\times 1,000}\times 100\%$$

### pH

The pH value of marinated beef steak was measured in triplicate after homogenizing 5 g of the sample (beef steak) with 45 ml of distilled water in a homogenizer (OHAUS a-AB23PH; OHAUS).

### Fatty acid profile

The fatty acid composition of the beef steak was determined using a gas chromatography/flame-ionization detection instrument (Clarus 690-IN 0264) [21]. Initially, 20 g of finely ground samples were extracted (Duplo) with a hexane solution.

Subsequently, 1.5 mL of hexane was added to the mixture of fatty acid methyl esters. One mL of the resulting mixture was taken and injected into the GC port using an autosampler. The split ratio used was 100:1 with an injector temperature of 250°C. The fatty acid methyl esters were then separated using a DB FastFAME fused silica capillary column with a helium flow of 1.0 mL/min. The fatty acids were identified by comparing the identified peaks with the retention times of the fatty acid standards (47,015-U; Sigma-Aldrich). The peaks observed in the chromatogram derived from the sample measurement results correspond to the peaks of C_4_–C_24_ fatty acids. These peaks are identified through a comparison of the retention time of each fatty acid component present in the sample with the retention time of each fatty acid component present in the standard. The calculation of fatty acid content is performed using the normalization method, whereby all fatty acid components present in the sample are represented in the chromatogram results, so that the total area under the peak represents 100% of the fatty acid content.

### Statistical analysis

Two-way ANOVA was used to analyze the data using PASW SPSS software. The means of interest were analyzed using the Tukey test at a 5% level of significance.

## RESULTS AND DISCUSSION

### Multilayer encapsulation of single-bulb garlic extract characterization

**Figure 1:**
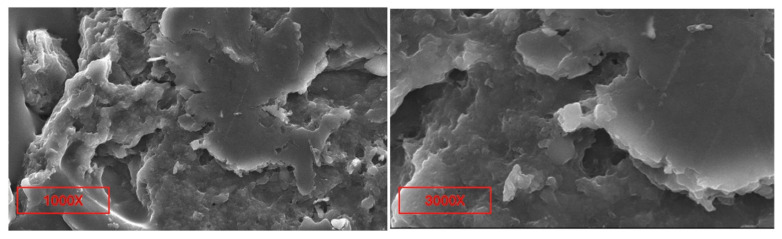
Scanning electron microscopy (SEM) images of multilayer encapsulation of single-bulb garlic extract (MESG): (A) 1,000 × and (B) 3,000 × magnification. Samples were freeze-dried for 48 h prior to analysis. Irregular particle morphology and surface roughness are observed.

**Figure 2:**
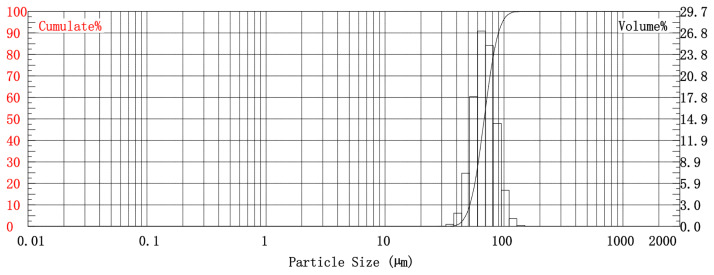
Particle size distribution of multilayer encapsulation of single-bulb garlic extract (MESG) determined by laser particle size analysis (LPSA). Bars represent volume-based distribution, and the solid line indicates cumulative volume percentage. Samples were freeze-dried for 48 h.

Figure 1 points out the SEM analysis results of MESG (A: 1,000×, B: 3,000×), along with the characteristics of the powder with a wavy, complex, and porous structure. There were also flakes or thin layers which overlapped or stuck to the surface, indicating the formation of layered or fragmented materials. Porosity in the encapsulated powder is a very important factor and generally has a positive effect on its dispersion ability. Powders with good porous structures, is easy to wet, absorb water quickly, and is evenly dispersed in the liquid. The hydrophilic properties of POL, which forms the outer layer of the encapsulation, will aid the dispersion process. A microstructure analysis pinpoints that adding POL promotes the entry of water molecules into the surface [22]. Additionally, the particle size was 66.4 μm (Figure 2), placing the product at a balance point between solubility rate and ease of handling, both are ideal properties for a powder intended for dissolution [23].

Nuraini et al [7] stated that single bulb garlic extract has a TPC value of 61.02 mg GAE/g, an antioxidant activity value (IC_50_) of DPPH of 10.35 ppm and a FRAP value of 94.1 μM equivalent to Fe(II)/g. Table 1 demonstrated comparison of the antioxidant activity values of the extract and MESG. Encapsulation protects sensitive antioxidant compounds from environmental factors such as light, heat and oxygen, which can degrade their activity.

### Free fatty acids

FFAs are fatty acids that are released from triglycerides (the main form of fat found in meat) through a process of enzymatic hydrolysis or chemical reactions. FFAs contribute to the sensory quality of meat, affecting its flavor, aroma, and texture. Although FFAs are present in low concentrations in fresh meat, their levels tend to increase over time due to lipid oxidation or microbial activity, resulting in rancidity and off-flavours [24]. The increase in FFAs content depends on the storage time for all treatments. Additionally, prolonged storage, along with the influences of temperature, pH, and the activity of enzymes and microorganisms, leads to the breakdown of triglycerides into FFAs and glycerol. FFA is analyzed to measure the decomposition values of triglycerides and phospholipids, which are present in meat during storage. In this study, the steak-sized cuts of meat indicated FFA levels ranging from 0.144±0.014 % to 0.371±0.018 % (Table 2). Increasing MESG in the meat significantly reduced FFA levels during storage (p<0.05), with the highest level of 0.371±0.018 % at MESG-0% on the 12 day of storage. Meanwhile, meat treated with encapsulated garlic marinade exhibited FFA levels which continued to rise until day 3 of storage, but then gradually declined.

The results of this study are consistent with previous research, pumpkin leaf ethanol extract to inhibit pork oxidation, where the addition of antioxidant compounds positively influenced FFA levels of the pork up to day 10 of storage. The best treatment exhibited an FFA value of 0.96% [25], which was higher compared to FFA value in this study. In addition to microbial factors, one of the most common causes of meat spoilage during storage is lipid oxidation, which results from lipid hydrolysis or the oxidation of polyunsaturated fatty acids (PUFAs). The addition of antioxidants is considered an effective preventive measure, because antioxidants will function as electron donors or proton donors, thereby reducing free radical activity and inhibiting lipid oxidation reactions. In this study, the application of multilayer encapsulation reduced FFA levels because antioxidants captured or neutralized free radicals before they could react with lipids in meat. Garlic contains organosulfur compounds such as allicin, alliin, and allyl disulfide, which have been shown to scavenge free radicals, thereby reducing oxidative stress [26]. This reduction in oxidative stress helps prevent the oxidation of lipids, including FFAs. The organosulfur compounds in garlic reduce the generation of reactive oxygen species (ROS), which are highly reactive molecules that can lead to the oxidation of FFAs [27]. By lowering ROS levels, garlic helps in maintaining the integrity of lipids.

### Antioxidant activity

Increasing the concentration of MESG significantly elevates antioxidant levels (p<0.05). The addition of MESG-8% maintained antioxidant activity until day 9 with an antioxidant activity value of 47.25±2.333 μM equivalent Fe(II)/g (Table 3), which was the highest compared to the MESG-4% and MESG-0%. Encapsulation helps maintain antioxidant activity during storage and keeps them stable and extend the shelf life. Encapsulation technologies are effective measures employed to protect against the degradation of bioactive substances.

Antioxidants stored in the encapsulated core are gradually released when they come into contact with the meat layer. Significant changes in pH will break down the encapsulation walls and release the antioxidants stored in the core. Antioxidant activity decreased over time during storage, but slightly increased on day 15 of storage. This is possible due to the presence of endogenous antioxidants in meat, which are formed as a result of protein degradation during storage. This is in line with the increase in meat pH during the 6 and 15 days of storage at MESG-0% (Table 4). The increase in meat pH indicates protein degradation in meat during storage. The breakdown of muscular beef proteins during chilled storage results in the generation of peptides with antioxidant activities [28]. Wu et al [29] stated different protein patterns and degradation rates of structural proteins were found depending on meat pHu. Large structural proteins, including myosin, undergo rapid changes within 48 hours post-mortem and show a rapid decline with storage time. Peptides from myosin were found to protect cells from oxidative damage by inhibiting ROS generation and activating endogenous antioxidant defense systems [30].

Protein degradation through enzymatic hydrolysis, involves the cleavage of peptide bonds, leading to the production of smaller peptide fragments. The antioxidant activity exhibited by these fragments depends on their amino acid composition and structural characteristics. Although peptide bonds do not directly form antioxidants, their cleavage during protein degradation releases bioactive peptide fragments with structural and chemical features which may generate antioxidant properties [31].

### Thiobarbituric acid reactive substances

Table 5 presents the changes of TBARS in beef steak-cuts during storage values ranging from 0.081±0.007 mg MDA/kg to a maximum of 0.593±0.052 MDA/kg. Statistical showed that TBARS value were significantly affected by storage time and the addition of MESG (p<0.05). Previous research stated that crushed garlic can reduce the percentage of beef fat and slow down the increase in TBARS during storage at refrigerator temperature [32]. The addition of 12% crushed garlic resulted in a TBARS value of 0.718 mg MDA/kg on the 12 day of storage, which was higher than in this study.

Table 1 reported that single-bulb garlic extract (Temanggung variety) has an antioxidant capacity of 94.14 μM equivalent Fe(II)/g supported by multilayer encapsulation that protects antioxidants from damage during storage. In this research, the addition of antioxidants reduced TBARS values until day 9 of storage, followed by a slight increase before decreasing again. This fluctuation might be a result of encapsulated antioxidant release, with the addition of MESG-8% generating the lowest TBARS value up to 15 days of storage. Meanwhile, in the research of Al-Rubeii et al [33] it was reported that the addition of 0.01% BHA/BHT to beef produced a TBARS value of 0.3 mg MDA/kg on day 9, which is higher than the addition of MESG-4% and MESG-8%, with TBARS values of 0.161±0.067 mg MDA/kg and 0.226±0.113 mg MDA/kg, respectively.

Natural antioxidants can inhibit oxidative damage [34], in this study, they support TBARS value reduction. heir inhibiting capacity is mediated through multiple mechanisms, such as direct radical neutralization, metal chelation, enzyme regulation, and synergistic interactions. Encapsulation contributes to this process by maintaining the stability of the active compounds during processing [14] which provide optimal protection against oxidative damage.

### pH

Meat pH indicates the level of acidity or alkalinity in meat tissue, which can provide important information about the quality and freshness of meat. In this research, the pH of beef loin samples ranged from 5.288±0.142 to 7.633±0.025 after day 15 of storage (Table 4). The addition of MESG stabilized the sample pH during refrigerated storage; thus, even though fluctuations, the pH remained acceptable. Meanwhile, the MESG-0% experienced a significant increase in pH, reaching 7.633±0.025 on day 15 of storage. Similar results were also found in previous research, where the pH of fresh pork patties containing 2.8% of fresh garlic was higher compared to other treatments (p<0.001) [35]. In this study, multilayer encapsulation using CH, arabic gum and POL began to be released on the 3 day of storage when the pH of the meat decreased towards a more acidic direction (Table 4) reinforced by the analysis of antioxidants which increased on 3 day of storage (Table 3). Naranjo-Durán et al [36] argued that encapsulation of bioactive compounds using alginate and CaCl_2_ achieved optimal release at pH 6.5. Meanwhile, a combination of leaf extracts encapsulated using GA showed controlled release under both acidic (pH of 2.2) and neutral (pH of 7.4) conditions [37].

### Fatty acid profile

Garlic reduces fatty acids in meat by inhibiting synthesis, increasing oxidation, and preventing oxidative loss. These effects improve the nutritional quality and stability of meat products. Four main fatty acids in back fat, namely palmitic acid (C16:0), stearic acid (C18:0), oleic acid (C18:1n9), and linoleic acid (LA) (C18:2n6), account for approximately 90% of the total fatty acids, with saturated fatty acids (SFAs) and monounsaturated fatty acids (MUFAs) dominating. Two types of unsaturated fatty acids protected by the marinating MESG treatment include C20:4 w6 (arachidonic acid) and C20:2 (eicosadienoic acid), which are both PUFAs. The findings of this study are consistent with those of previous research, which demonstrated that levels of arachidic acid increased and palmitoleic acid decreased in the group given garlic supplements [38]. Garlic enhances the oxidation of fatty acids, which helps in reducing their levels in meat. Table 6 shows the changes in fatty acids during storage, both saturated and unsaturated fatty acids, and there was a decrease in some of these acids (p<0.05).

In this research, the addition of MESG-4% and MESG-8% caused a decrease in LA levels compared to MESG-0% as well as a significant decrease in SFAs compared to untreated samples. Garlic compounds such as allicin suggest activate AMP-activated protein kinase (AMPK). Based on an understanding of existing literature, protection against oxidation is achieved through fatty acids reduction mechanisms, with AMPK/PPAR-α activation reducing lipolysis, thereby minimizing the release of FFAs susceptible to oxidation. During the pre-rigor phase, ATP is still being metabolized through anaerobic glycolysis. AMPK can be transiently activated due to an increase in the AMP/ATP ratios, which affects the rate of glycogen breakdown and glycolysis [39]. This brief activity might indirectly affect lipid metabolic pathways if fatty acid oxidation continues. The presence of LA in substrate has been observed to promote lipoxygenase (LOX) and cyclooxygenase (COX) activity, which then convert them to hydroperoxides or prostaglandins. Garlic extract has been suggested to enhance LOX activity in a short-lived manner in the early post-mortem stage, thereby accelerating LA breakdown. DAS protects PUFAs in the bilayer membrane by inhibiting the LOX/MPO enzymes, thereby preventing PUFA oxidation [40]. The findings indicated MESG functions as a protective agent against damage up to 15 days of refrigerated storage, potentially due to its antioxidant content, which may help preserve PUFAs.

## CONCLUSION

The formulation of MESG resulted in the establishment of a porous structure and hydrophilic properties. This combination then allowed for dispersion and controlled release of bioactive compounds. MESG inhibited oxidative damage in beef by preserving a stable fatty acid profile and resulting in significantly lower FFA levels and TBARS values after 15 days of storage. Nevertheless, several limitations should be noted in this study. The absence of a direct comparison between encapsulated and non-encapsulated garlic extracts is notable. This hampers conclusive quantification of specific protective improvements due to encapsulation processes, such as elevated stability, targeted delivery, and longer efficacy, based on the current data.

## Figures and Tables

**Table 1:** Comparison between the antioxidant activity (μM Fe(II) equivalents/g) of single-bulb garlic extract and MESG

**Antioxidant activity**	**Single-bulb garlic extract**	**MESG**

	94.14±10.18^1)^	101±0.10

Values are expressed as mean±SD (n = 3).

1) Observational data reproduced from Nuraini et al [7].

MESG, multilayer encapsulation of single-bulb garlic extract; SD, standard deviation.

**Table 2:** The concentration of FFA (%) in beef steak stored at refrigerator temperature for 15 d (observed every 3 d)

Storage time (d)	Treatment^1)^		

	MESG-0%	MESG-4%	MESG-8%
0	0.205±0.001^Ac^	0.208±0.006^Ab^	0.205±0.001^Aa^
3	0.256±0.001^Bc^	0.251±0.005^Bb^	0.238±0.010^Ba^
6	0.282±0.001^Bc^	0.235±0.018^Bb^	0.224±0.016^Ba^
9	0.367±0.007^Bc^	0.179±0.005^Bb^	0.193±0.035^Ba^
12	0.371±0.018^Bc^	0.205±0.001^Bb^	0.166±0.011^Ba^
15	0.305±0.004^Ac^	0.177±0.004^Ab^	0.144±0.014^Aa^

Values are expressed as mean±SD (n = 3).

1) MESG-0%, MESG-4%, and MESG-8% represent beef samples marinated with 0%, 4%, and 8% multilayer single-bulp garlic extract, respectively.

A,B Means in a same row with different superscripts differ significantly (p<0.05).

a–c Means in a same column with different superscripts differ significantly (p<0.05).

FFA, free fatty acid, MESG, multilayer encapsulation of single-bulb garlic extract; SD, standard deviation.

**Table 3:** Antioxidant activity (μM equivalent Fe(II)/g) in beef steak stored at refrigerator temperature for 15 d (observed every 3 d)

Storage time (d)	Treatment^1)^		

	MESG-0%	MESG-4%	MESG-8%
0	25.116±0.115^Ea^	26.616±1.328^Eb^	25.5±0.086^Ec^
3	34.75±1.060^Fa^	68.383±3.957^Fb^	61.35±0.001^Fc^
6	2.00±0.173^Da^	33.8±0.001^Db^	34.15±1.060^Dc^
9	4.2±0.001^Ca^	10.67±3.763^Cb^	47.25±2.333^Cc^
12	4.05±0.001^Ba^	17.75±0.001^Bb^	18.80±0.070^Bc^
15	10.8±0.436^Aa^	10.1±0.346^Ab^	9.667±0.153^Ac^

Values are expressed as mean±SD (n = 3).

1) MESG-0%, MESG-4%, and MESG-8% represent beef samples marinated with 0%, 4%, and 8% multilayer single-bulp garlic extract, respectively.

A–F Means in a same row with different superscripts differ significantly (p<0.05).

a–c Means in a same column with different superscripts differ significantly (p<0.05).

MESG, multilayer encapsulation of single-bulb garlic extract; SD, standard deviation.

**Table 4:** pH in beef steak stored at refrigerator temperature for 15 d (observed every 3 d)

Storage time (d)	Treatment^1)^		

	MESG-0%	MESG-4%	MESG-8%
0	5.847±0.050^Bc^	5.78±0.121^Bb^	6.097±0.064^Bc^
3	5.42±0.173^Ac^	5.365±0.067^Ab^	5.288±0.142^Ac^
6	6.43±0.204^CDc^	6.482±0.215^CDb^	6.127±0.176^CDc^
9	6.537±0.226^CDc^	6.488±0.159^CDb^	6.455±0.117^DEc^
12	5.905±0.001^Cc^	6.065±0.303^Cb^	6.518±0.029^Cc^
15	7.633±0.025^Ec^	6.32±0.448^Eb^	5.993±0.606^Ec^

Values are expressed as mean±SD (n = 3).

1) MESG-0%, MESG-4%, and MESG-8% represent beef samples marinated with 0%, 4%, and 8% multilayer single-bulp garlic extract, respectively.

A–E Means in a same row with different superscripts differ significantly (p<0.05).

a–c Means in a same column with different superscripts differ significantly (p<0.05).

MESG, multilayer encapsulation of single-bulb garlic extract; SD, standard deviation

**Table 5:** The concentration of TBARS (mg MDA/kg) in beef steak stored at refrigerator temperature for 15 d (observed every 3 d)

Storage time (d)	Treatment^1)^		

	MESG-0%	MESG-4%	MESG-8%
0	0.092±0.001^Ab^	0.121±0.012^Aa^	0.139±0.006^Aa^
3	0.449±0.001^Bb^	0.081±0.007^Ba^	0.182±0.052^Ba^
6	0.593±0.052^Cb^	0.137±0.032^Ca^	0.252±0.013^Ca^
9	0.459±0.001^Bb^	0.161±0.067^Ba^	0.226±0.113^Ba^
12	0.324±0.001^Bb^	0.234±0.001^Ba^	0.244±0.014^Ba^
15	0.324±0.001^Bb^	0.361±0.099^Ba^	0.169±0.037^Ba^

Values are expressed as mean±SD (n = 3).

1) MESG-0%, MESG-4%, and MESG-8% represent beef samples marinated with 0%, 4%, and 8% multilayer single-bulp garlic extract, respectively.

A–C Means in a same row with different superscripts differ significantly (p<0.05).

a,b Means in a same column with different superscripts differ significantly (p<0.05).

TBARS, thiobarbituric acid reactive substances; MESG, multilayer encapsulation of single-bulb garlic extract; SD, standard deviation.

**Table 6:** Comparison of fatty acid profile content in MESG-0%, MESG-4% and MESG-8% stored for 15 d at refrigerated temperature

Fatty acid	Treatment^1)^			

	MESG-0%	MESG-0%	MESG-4%	MESG-8%

	0 d (control)	12 d	12 d	12 d
Omega 3	0.024±0.0001^d^	0.0128±0.0001^c^	0.0045±0.0001^a^	0.0094±0.0001^b^
Omega 6	0.1053±0.0001^d^	0.0378±0.0002^c^	0.0165±0.0002^a^	0.035±0.0001^b^
Omega 9	0.5366±0.0009^d^	0.4244±0.0009^c^	0.1535±0.0008^b^	0.1053±0.0008^a^
Linoleic acid	0.0905±0.0004^d^	0.0339±0.0001^c^	0.0155±0.000^a^	0.0309±0.000^b^
Linolenic acid	0.0118±0.0002^c^	0.0065±0.0001^b^	ND	0.0025±0.000^a^
Oleic acid	0.5366±0.0009^d^	0.4244±0.0009^c^	0.1535±0.0008^b^	0.1053±0.0008^a^
C14:0	0.0301±0.0002^c^	0.0418±0.0004^d^	0.0089±0.0001^b^	0.0056±0.0001^a^
C15:0	0.0099±0.0002^c^	0.0058±0.0001^b^	0.0036±0.0001^a^	ND
C16:0	0.4006±0.0010^d^	0.2935±0.0003^c^	0.1056±0.0004^b^	0.0806±0.0004^a^
C16:1	0.0226±0.0003^d^	0.0181±0.0007^c^	0.0063±0.0001^b^	0.0051±0.0001^a^
C17:0	0.0178±0.0004^d^	0.0163±0.0001^c^	0.0056±0.0001^b^	0.0028±0.0001^a^
C17:1	0.0067±0.000^b^	0.0051±0.000^a^	ND	ND
C18:0	0.2831±0.0010^d^	0.2375±0.0004^c^	0.0875±0.0002^b^	0.0454±0.0009^a^
C18:1 ω-9C	0.5365±0.0009^d^	0.4244±0.0009^c^	0.1535±0.0008^b^	0.1053±0.0008^a^
C18:2 ω-6	0.0905±0.0004^c^	0.0339±0.0004^b^	ND	0.0309±0.0001^a^
C18:2 ω-6C	0.0905±0.0004^d^	0.0339±0.0001^c^	0.0154±0.0001^a^	0.0309±0.0001^b^
C18:3 ω-3	0.010±0.0001^c^	0.0045±0.0001^b^	0.0154±0.0001^d^	0.0025±0.0001^a^
C20:0	0.0033±0.000^a^	0.00285±0.000^b^	ND	ND
C20:2	0.0060±0.000^c^	0.003±0.000^b^	0.0027±0.000^a^	0.0066±0.0001^c^
C20:4 ω-6	0.013±0.000^b^	ND	ND	0.0041±0.000^b^
DHA	0.009±0.0001^d^	0.0082±0.0001^c^	0.0029±0.0001^a^	0.0068±0.0001^b^
SFA	0.7475±0.0009^d^	0.6023±0.0004^c^	0.2131±0.0005^b^	0.1367±0.0006^a^
UFA	0.702±0.0009^d^	0.5076±0.0004^c^	0.1869±0.0005^b^	0.1632±0.0006^a^
PUFA	0.13530±0.0005^d^	0.05355±0.0002^b^	0.02365±0.0002^a^	0.05105±0.000^c^
MUFA	0.5671±0.0010^d^	0.4541±0.0007^c^	0.1633±0.0008^b^	0.1121±0.0006^a^

Values are expressed as mean±SD (n = 3).

1) MESG-0%, MESG-4%, and MESG-8% represent beef samples marinated with 0%, 4%, and 8% multilayer single-bulp garlic extract, respectively.

a–d Means in a same column with different superscripts differ significantly (p<0.05).

MESG, multilayer encapsulation of single-bulb garlic extract; ND, not detected; values below the detection limit (<0.001); DHA, docosahexaenoic acid; SFA, saturated fatty acids; UFA, unsaturated fatty acids; PUFA, polyunsaturated fatty acids; MUFA, monounsaturated fatty acids; SD, standard deviation.

## Data Availability

Upon reasonable request, the datasets of this study can be available from the corresponding author.

## References

[1] de Carvalho FAL Lorenzo JM Pateiro M Bermúdez R Purriños L Trindade MA Effect of guarana (Paullinia cupana) seed and pitanga (Eugenia uniflora L.) leaf extracts on lamb burgers with fat replacement by chia oil emulsion during shelf life storage at 2°C Food Res Int 2019 125 108554 10.1016/j.foodres.2019.108554 31554074

[2] Ribeiro JS Santos MJMC Silva LKR Natural antioxidants used in meat products: a brief review Meat Sci 2019 148 181 8 10.1016/j.meatsci.2018.10.016 30389412

[3] Cunha LCM Monteiro MLG Costa-Lima BRC Effect of microencapsulated extract of pitaya (Hylocereus costaricensis) peel on color, texture and oxidative stability of refrigerated ground pork patties Submitted to high pressure processing Innov Food Sci Emerg Technol 2018 49 136 45 10.1016/j.ifset.2018.08.009

[4] Bellucci ERB Bis-Souza CV Domínguez R Bermúdez R da Silva Barretto AC Addition of natural extracts with antioxidant function to preserve the quality of meat products Biomolecules 2022 12 1506 10.3390/biom12101506 36291715 PMC9599661

[5] Fadhil KA Suryati T Jayanegara A Comparison between natural and synthetic antioxidants in beef products: a meta analysis J I Produksi Teknol Hasil Peternakan 2023 11 19 26 10.29244/jipthp.11.1.19-26

[6] Nikmaram N Budaraju S Barba FJ Application of plant extracts to improve the shelf-life, nutritional and health-related properties of ready-to-eat meat products Meat Sci 2018 145 245 55 10.1016/j.meatsci.2018.06.031 29982079

[7] Nuraini V Legowo AM Mulyani S Adiwinarti R Exploration antioxidant properties of Indonesian local single-bulb garlic extract (Var. Temanggung) in a mixed solvent J Food Sci Technol 2025 22 35 49 10.22034/FSCT.22.160.35

[8] McClements DJ Li F Xiao H The nutraceutical bioavailability classification scheme: classifying nutraceuticals according to factors limiting their oral bioavailability Annu Rev Food Sci Technol 2015 6 299 327 10.1146/annurev-food-032814-014043 25705933

[9] Agustin DA Wibowo AA Encapsulation technology: techniques and applications Distilat J Teknol Sep 2021 7 202 9 10.33795/distilat.v7i2.210

[10] Steiner BM McClements DJ Davidov-Pardo G Encapsulation systems for lutein: a review Trends Food Sci Technol 2018 82 71 81 10.1016/j.tifs.2018.10.003

[11] Meng Q Zhong S Wang J Gao Y Cui X Advances in chitosan-based microcapsules and their applications Carbohydr Polym 2023 300 120265 10.1016/j.carbpol.2022.120265 36372516

[12] Huang S Liu J Li G Liu Y Huang B Li J Effect of wall material on the structure and antioxidant activities of bamboo leaves flavonoid microcapsules Sci Technol Food Ind 2021 42 55 61 10.13386/j.issn1002-0306.2021020043

[13] Kuhn F de Azevedo ES Noreña CPZ Behavior of inulin, polydextrose, and egg albumin as carriers of Bougainvillea glabra bracts extract: rheological performance and powder characterization J Food Process Preserv 2020 44 e14834 10.1111/jfpp.14834

[14] Rezagholizade-shirvan A Soltani M Shokri S Radfar R Arab M Shamloo E Bioactive compound encapsulation: characteristics, applications in food systems, and implications for human health Food Chem X 2024 24 101953 10.1016/j.fochx.2024.101953 39582652 PMC11584689

[15] Wan J Pei Y Hu Y Microencapsulation of eugenol through gelatin-based emulgel for preservation of refrigerated meat Food Bioprocess Technol 2020 13 1621 32 10.1007/s11947-020-02502-0

[16] Tavares L Noreña CPZ Characterization of the physicochemical, structural and thermodynamic properties of encapsulated garlic extract in multilayer wall materials Powder Technol 2021 378 388 99 10.1016/j.powtec.2020.10.009

[17] Komala PTH Husni A Extraction temperature effect on methanolic extract antioxidant activity of Eucheuma spinosum J Pengolah Has Perikan Indones 2021 24 1 10 10.17844/jphpi.v24i1.34193

[18] Houghton JE Behnsen J Duller RA Nichols TE Worden RH Particle size analysis: a comparison of laboratory-based techniques and their application to geoscience Sediment Geol 2024 464 106607 10.1016/j.sedgeo.2024.106607

[19] Katsanidis E Zampouni K Development of a novel steam distillation TBA test for the determination of lipid oxidation in meat products Foods 2023 12 359 10.3390/foods12020359 36673451 PMC9857627

[20] Barido FH Jang A Pak JI Kim YJ Lee SK Combined effects of processing method and black garlic extract on quality characteristics, antioxidative, and fatty acid profile of chicken breast Poult Sci 2022 101 101723 10.1016/j.psj.2022.101723 35172234 PMC8851260

[21] Ratnayake WMN Hansen SL Kennedy MP Evaluation of the CP-Sil 88 and SP-2560 GC columns used in the recently approved AOCS official method Ce 1h-05: determination of cis-, trans-, saturated, monounsaturated, and polyunsaturated fatty acids in vegetable or non-ruminant animal oils and fats by capillary GLC method J Am Oil Chem Soc 2006 83 475 88 10.1007/s11746-006-1230-y

[22] Wang M Liu C Luo X Wu J Li X Effect of polydextrose on the cooking and gelatinization properties and microstructure of Chinese early indica rice Gels 2025 11 171 10.3390/gels11030171 40136876 PMC11942580

[23] Lai S Cui Q Sun Y Liu R Niu Y Effects of particle size distribution on the physicochemical, functional, and structural properties of alfalfa leaf powder Agriculture 2024 14 634 10.3390/agriculture14040634

[24] Soenarno MS Arifin M Prabowo S Evaluation of acid value, free fatty acids, and malondialdehyde (MDA) content in Chevon fat: a pre- and post-roasting comparison J Anim Prod Process Technol 2024 12 101 4 10.29244/jipthp.12.2.101-104

[25] Choe JH Kim HY Choi YS Effects of pumpkin (Cucurbita moschata Duch.) leaf ethanolic extracts on lipid oxidation and microbial activity in refrigerated raw ground pork Korean J Food Sci Anim Resour 2011 31 865 71 10.5851/kosfa.2011.31.6.865

[26] Locatelli DA Nazareno MA Fusari CM Camargo AB Cooked garlic and antioxidant activity: correlation with organosulfur compound composition Food Chem 2017 220 219 24 10.1016/j.foodchem.2016.10.001 27855892

[27] Dvořáková M Weingartová I Nevoral J Němeček D Krejčová T Garlic sulfur compounds suppress cancerogenesis and oxidative stress: a review Sci Agric Bohem 2015 46 65 72 10.1515/sab-2015-0018

[28] Mora L Bolumar T Heres A Toldrá F Effect of cooking and simulated gastrointestinal digestion on the activity of generated bioactive peptides in aged beef meat Food Funct 2017 8 4347 55 10.1039/c7fo01148b 28990613

[29] Wu G Farouk MM Clerens S Rosenvold K Effect of beef ultimate pH and large structural protein changes with aging on meat tenderness Meat Sci 2014 98 637 45 10.1016/j.meatsci.2014.06.010 25089788

[30] Chen J Yan Y Zhang L Purification of novel antioxidant peptides from myofibrillar protein hydrolysate of chicken breast and their antioxidant potential in chemical and H2O2-stressed cell systems Food Funct 2021 12 4897 908 10.1039/d1fo00579k 34100502

[31] Zhu Y Lao F Pan X Wu J Food protein-derived antioxidant peptides: molecular mechanism, stability and bioavailability Biomolecules 2022 12 1622 10.3390/biom12111622 36358972 PMC9687809

[32] Nurwantoro Bintoro VP Legowo AM Purnomoadi A Setiani BE Garlic antioxidant (Allium Sativum L.) to prevent meat rancidity Procedia Food Sci 2015 3 137 41 10.1016/j.profoo.2015.01.014

[33] Al-Rubeii AMS Al-Kaisey MT Khadom MJ Effect of some natural and synthetic antioxidants on ground beef meat during cold storage Alex J Food Sci Technol 2009 6 1 16

[34] Barbosa ACS Mendes PS Mattos G Comparative analysis of the use of natural and synthetic antioxidants in chicken meat: an update review Braz J Biol 2023 83 e275539 10.1590/1519-6984.275539 37878961

[35] Park SY Chin KB Effect of fresh garlic on lipid oxidation and microbiological changes of pork patties during refrigerated storage Korean J Food Sci Anim Resour 2014 34 638 46 10.5851/kosfa.2014.34.5.638 26761498 PMC4662226

[36] Naranjo-Durán AM Quintero-Quiroz J Rojas-Camargo J Ciro-Gómez GL Modified-release of encapsulated bioactive compounds from annatto seeds produced by optimized ionic gelation techniques Sci Rep 2021 11 1317 10.1038/s41598-020-80119-1 33446706 PMC7809057

[37] Almayda N Masruri M Safitri A Effectiveness of using gum Arabic for co-microencapsulation of Ruellia tuberosa L. and Tithonia diversifolia extracts as encapsulating agent and release studies Scientifica 2024 2024 9097238 10.1155/2024/9097238 38827017 PMC11142852

[38] Leung HH Yau YF Leung KS Garlic supplementation modified enzymatic omega-6 polyunsaturated fatty acid oxidation in mild hypercholesterolemia Eur J Lipid Sci Technol 2019 121 1900069 10.1002/ejlt.201900069

[39] Zhao X Wu S Ren C Revealing the mechanism of protein degradation in postmortem meat: the role of phosphorylation and ubiquitination Foods 2025 14 184 10.3390/foods14020184 39856851 PMC11764534

[40] Du M Zhao K Chen A Inhibitory mechanisms of lipoxygenase by garlic sulfides: “adsorption-embolism” effect of DAS and DADS and “quasi-domain-separation” effect of DATS and Allicin Int J Biol Macromol 2025 304 140874 10.1016/j.ijbiomac.2025.140874 39933676

